# Effect of a yeast autolysate produced by high pressure homogenization on white wine evolution during ageing

**DOI:** 10.1007/s13197-020-04867-8

**Published:** 2020-10-24

**Authors:** Sabrina Voce, Sonia Calligaris, Piergiorgio Comuzzo

**Affiliations:** Dipartimento di Scienze Agroalimentari, Ambientali e Animali, Università Degli Studi Di Udine, via Sondrio 2/A, 33100 Udine, Italy

**Keywords:** Yeast derivatives, HPH, Wine ageing, Aroma compounds, Browning, Glutathione

## Abstract

The enological characteristics and the performances of a yeast autolysate produced by high pressure homogenization (HPH-YD) were investigated for the first time in white wine and model solution, in comparison with a thermolysate (T-YD) and a commercial yeast derivative (COMM). In wine-like medium, HPH-YD showed a significant release of glucidic colloids (on average, slightly higher than the other products), also leading to a greater glutathione solubilization with respect to T-YD. Concerning the volatile composition of the autolysates, HPH-YD was characterized by the highest concentration of alcohols and esters, while showing an average amount of fatty acids, carbonyls and heterocyclic compounds lower than COMM. These features are potentially linked to a more favorable impact of this product on the composition of wine aroma, should these compounds be released into the wine itself. HPH-YD determined minor modifications on wine volatile profile when added for short contact times, without releasing unwanted compounds and with a slightly lower binding capacity towards wine esters. The effects of the three yeast derivatives (YDs) on wine color during ageing was also investigated in comparison with sulfur dioxide (SO_2_). HPH-YD was the most efficient preparation, limiting wine color changes due to oxidation during four months and behaving more similarly to SO_2_. The use of HPH for the production of yeast autolysates for winemaking may represent an interesting alternative to thermal treatments, improving the enological characteristics of these additives, particularly their antioxidant capacity, leading anyhow a significant release of colloidal molecules and a limited impact on wine aroma composition.

**Electronic supplementary material:**

The online version of this article (10.1007/s13197-020-04867-8) contains supplementary material, which is available to authorized users.

## Introduction

High pressure homogenization (HPH) technology has been explored in several areas of food industry, demonstrating great potential in microbial inactivation (Donsì et al. 2009; Patrignani and Lanciotti 2016) and modification of functional properties of food components, such as proteins (Yang et al. 2018), enzymes (dos Santos Aguilar et al. 2018) and polysaccharides (Spotti and Campanella 2017). All the effects attributed to HPH can be associated with the intense mechanical stresses suffered by the product during HPH process. In particular, during HPH the fluid is pumped through a narrow gap valve utilizing a pressure intensifier. The product eventually undergoes intense mechanical forces and elongational stresses at the valve entrance and in the valve gap, while turbulence, cavitation and impacts with the solid surface occur at the gap outlet (Floury et al. 2004). Different processing parameters (operating pressure, sample temperature, number of passes through the homogenization valve), as well as product characteristics (e.g. pH, composition, viscosity) could affect the effectiveness of HPH process (Donsì et al. 2009).

The exploitation of the disruptive forces delivered during HPH was recently proposed also for winemaking application. In particular, due to its ability to promote yeast cell breakdown (Liu et al. 2016), HPH was tested as efficient tool for accelerating yeast autolysis (Patrignani et al. 2013) or for obtaining autolyzed yeast derivatives (YDs) (Verduyn et al. 1999). The latter application is particularly interesting because of the wide utilization of such additives at winery scale.

YDs (inactive dry yeasts and yeast autolysates) for winemaking use are industrially obtained from enzymatic or thermally assisted cell lysis of *Saccharomyces*. The resulting products are characterized by a heterogeneous composition, consisting of soluble and insoluble components in musts and wines. YD products are claimed to have several possible applications in wine industry: supplying of soluble molecules and polysaccharides (Pozo-Bayón et al. 2009a), interaction with aroma compounds modulating their volatility (Pozo-Bayón et al. 2009c; Comuzzo et al. 2011), improvement of wine sensory characters (Pozo-Bayón et al. 2009c), as well as release of antioxidant molecules (Rodríguez-Bencomo et al. 2014; Comuzzo et al. 2015a). In addition, YDs are commonly used also as fermentation enhancers, particularly to prevent the risk of stuck and sluggish fermentations (Edward and Beelman 1987; Bisson and Butzke 2000).

Besides their positive functionalities in wine, commercial YDs may sometimes negatively affect wine sensory characteristics by releasing off-flavors (Comuzzo et al. 2006; Pozo-Bayón et al. 2009b). These unpleasant compounds may be formed in commercial products during YD manufacturing. The most common technologies for the production of YDs are based on the induction of autolytic enzymes (Nagodawithana 1992) or thermolytic methods (Middelberg 1995). Both these approaches may give rise to strong impact odorants in the commercial preparations, the former because of the excessive protein degradation and the subsequent formation of aroma precursors (amino acids) (Charpentier and Feuillat 1993), the latter for the high processing temperatures achieved during manufacturing (Münch et al. 1997).

Recently, HPH was proposed as unconventional alternative technology for the production of YDs for winemaking use (Comuzzo et al. 2015b, 2017). It has been demonstrated that, upon HPH application at 150 MPa for increasing passes, yeast cell breakdown can be obtained at temperatures lower than those applied in conventional thermally assisted yeast cell lysis. In such operating conditions, certain HPH-YDs released a good concentration of glucidic colloids in wine-like medium, low amounts of proteins and amino acids, being also characterized by low levels of possible off-flavors that could negatively affect wine sensory properties (Comuzzo et al. 2017). However, currently, no scientific evidences are available concerning the modifications induced in wine by the supplementation with HPH-YDs. Therefore, a validation on a real matrix is needed to better elucidate the potential benefits of this technology at winery scale.

This work studied the enological performance of a yeast autolysate produced by high pressure homogenization (HPH-YD) in comparison with a yeast thermolysate produced at lab-scale (T-YD) and a commercial YD preparation specifically marketed for winemaking use (COMM). The three YDs were firstly characterized for their volatile composition by SPME–GC–MS. Furthermore, their ability to release soluble compounds, such as glucidic colloids, amino acids and glutathione in wine-like medium was studied. Finally, the effects of the HPH-YD were investigated in white wine for the first time, in comparison with the other two YD preparations, considering wine volatile profile, color changes and predisposition to browning, after one and four months of storage.

## Materials and methods

### Yeast derivatives

The commercial YD preparation (COMM) was purchased from a local supplier. It was a yeast derivative specifically marketed for winemaking application, described as a product obtained by thermal inactivation of a high-glutathione producing *Saccharomyces* strain. The YDs obtained by thermolysis (T-YD) and HPH treatment (HPH-YD) were produced in laboratory from the same commercial active dry yeast preparation (ADY), *Saccharomyces bayanus* (Mycoferm Cru-05), supplied by EverIntec (Pramaggiore, Venice, Italy).

Thermolysis for T-YD preparation was carried out by suspending 20 g of ADY in 200 mL of MilliQ grade water followed by thermal treatment in autoclave at 121 °C for 2 h.

HPH-YD was obtained by using a Panda PLUS 2000 two stage high pressure homogenizer (Gea Niro Soavi, Parma, Italy), provided with two cylindrical tungsten carbide homogenizing valves. Aliquots of 40 g of ADY preparation were suspended in 400 mL of Milli Q grade water and processed at a pressure of 150 MPa, for 10 passes, operating at a flow rate of 10 L h^−1^. Sample temperature was measured at the homogenizer inlet and outlet; the values ranged from 23 (inlet) to 74 °C (outlet, after the 10^th^ pass). These operating conditions were selected to obtain a product, in agreement with the recommendations of International Organization of Vine and Wine (OIV) regarding yeast-derived products for winemaking: the maximum amount of viable cells in inactive yeasts and yeast autolysates should be lower than 10^2^ CFU g^−1^ (International Organization of Vine and Wine 2017). After 10 passes at 150 MPa, the number of viable cells grown on Malt-Extract Agar was lower than 10 CFU g^−1^.

After HPH and thermolysis treatments, the suspensions were collected in food-grade aluminum trays (approx. in a 1 cm layer), frozen at -20 °C and freeze-dried by a Mini Fast 1700 freeze-dryer (Edwards Alto Vuoto, Milan, Italy). The samples were ground to obtain a powder and stored at 0/ + 4 °C, until further chemical and volatile analyses. All samples were subjected to the analytical determinations reported below.

### Analytical determinations on yeast derivatives

#### Release of glucidic colloids in wine-like medium

One of the most interesting aspects connected with the use of YDs during wine ageing, is their ability to release soluble colloids and polysaccharides in wine (Pozo-Bayón et al. 2009a). Aliquots of 1 g of the freeze-dried powders were suspended in 10 mL of wine-like medium (hydroalcoholic-tartaric buffer prepared with 5 g L^−1^ tartaric acid and 12% v/v ethanol, pH 3.20). After 10 min, the suspensions were centrifuged (10 min, 5000 rpm); 5 mL of the supernatant were mixed with 25 mL of ethanol (96% v/v) and kept at 0/ + 4 °C for 24 h. Glucidic colloids were separated by vacuum filtration on 0.45 µm cellulose acetate membranes (Albet-Hahnemühle, Barcelona, Spain) and quantified by weighing after complete evaporation of ethanol (at 50 °C, until constant weight). Data were given in mg of total colloids per g of freeze-dried powder.

#### Release of amino acids in wine-like medium

The release of amino acids is another characteristic claimed for YD products. This feature may be very interesting when such preparations are supplemented on grape juice as fermentation enhancers, but it may increase the risk of microbiological instability when YDs are used for wine ageing.

Amino acids were quantified spectrophotometrically by *o*-phthaldialdehyde (OPA) derivatization, according to the method published by Dukes and Butzke (Dukes and Butzke 1998). Aliquots of 0.10 g of freeze-dried powders were suspended in 10 mL of hydroalcoholic-tartaric buffer (pH 3.20, ethanol 12% v/v). After 10 min, the suspensions were centrifuged (10 min, 5000 rpm) and the supernatant was subjected to OPA assay as reported by the authors (Dukes and Butzke 1998).

#### Release of glutathione in wine-like medium

Glutathione (GSH) is considered one of the most powerful antioxidant molecules among the components of YDs; it is well known that GSH may protect must and wine polyphenols against oxidation, also preserving certain wine aroma compounds (e.g. esters, terpenes and volatile thiols) during wine ageing (Rodríguez-Bencomo et al. 2014).

For GSH determination, aliquots of 0.10 g of YD powder were suspended in 10 mL of hydroalcoholic-tartaric buffer (pH 3.20, ethanol 12% v/v). After 10 min, the suspensions were centrifuged (10 min, 5000 rpm) and 20 µL of supernatant were introduced in 5 mm optical path length glass cuvettes. Total GSH was determined spectrophotometrically, by the enzymatic method described by Adams and Liyanage (Adams and Liyanage 1991).

#### Volatile composition of the freeze-dried powders

The volatile composition of the YD powders was characterized by SPME–GC–MS, as reported previously (Comuzzo et al. 2015b). Analyses were carried out using a GC-17A gas chromatograph equipped with a QP-5000 mass spectrometer (Shimadzu, Kyoto, Japan). Autolysate samples (2.00 g) were introduced in 50 mL amber glass vials sealed with PTFE/silicone septa. Vials were pre-conditioned for 15 min at 40 °C before microextraction, and SPME was run at the same temperature for 15 min, using a 2 cm 50/30 µm divinylbenzene/carboxen/polydimethylsiloxane fiber (Supelco, Bellefonte, PA, USA). A J&W DB-Wax capillary column, 30 m × 0.25 mm i.d., 0.25 µm film thickness (Agilent Technologies Inc., Santa Clara, CA, USA) was used for the GC separation, with the following operating conditions: 40 °C for 1 min, then 4 °C min^−1^, up to 240 °C, with a final holding of time of 15 min. Injection was performed in splitless mode with 60 s of splitless time; injection port and transfer line were set at 250 and 240 °C respectively. Carrier gas was helium, at a linear flow rate of 35 cm s^−1^.

### Assessment of YDs in the white wine samples

The enological performances of the three yeast derivatives (COMM, T-YD and HPH-YD) were tested on a white wine, a Chardonnay D.O.C. Grave del Friuli (harvest 2013, alcoholic strength 12.14% v/v, free SO_2_ 10 mg L^−1^ and pH 3.35), supplied by Viticoltori Friulani “La Delizia” (Casarsa della Delizia, Pordenone, Italy).

Two series of samples were prepared. The former was used for aroma analysis: the wine was partitioned in 0.75 L glass bottles and aliquots of 0.4 g of the three YDs were added. Treatments were compared with a control sample, prepared without YD supplementation.

The second series of samples was prepared for color measurements and browning assay. It is well known that wine color changes during ageing; browning in white wine is induced by the oxidation of phenolic compounds into *o*-quinones, determined by too high oxygen transfer through the bottle closures (Lopes et al., 2009). Sulfur dioxide is the most efficient tool available for reducing the extent of browning reactions, because of its capacity to react with quinones reducing them back to polyphenols (Danilewicz et al., 2008); however, certain YDs demonstrated good antioxidant properties, being able to protect white wine color during bottle ageing (Comuzzo et al. 2015a). For this second set of samples, wine was introduced into 0.75 L glass bottles and saturated with oxygen by vigorous shaking. Oxygen level was checked by pasting onto the inner wall of the bottles an oxygen sensitive sensor (O2xyDot®, OxySense Inc., Dallas, TX, USA) and measuring the quenching of its fluorescence by an OxySense® fluorimeter (OxySense Inc., Dallas, TX, USA), as described previously (Comuzzo et al. 2015a). YDs were added to the oxygen saturated wines (0.4 g L^−1^) and samples were compared with a control test prepared by supplementation with 50 mg L^−1^ of sulfur dioxide (Control-SO_2_).

For both the series of bottles, nitrogen was blown in the headspace to eliminate oxygen before closing; samples were then sealed with crown cap closures, carefully homogenized and stored at 20 °C until analysis.

### Analytical determinations on YD supplemented wine samples

#### Wine color and browning assay

To assess eventual differences connected with the claimed ability of YDs to protect wine from browning, wine color was analyzed after one and four months, by measuring the absorbance of the samples at 420 nm. Wines were introduced in 10 mm optical path length glass cuvettes and absorbance was read against Milli Q grade water.

The predisposition of the treated wines towards browning was evaluated as previously described (Comuzzo et al. 2015a), using the POM-test, developed by Müller-Späth (Müller-Späth 1992). Both color measurements and browning assay were determined after filtration of wine samples on 0.45 µm cellulose acetate membranes (Albet-Hahnemühle, Barcelona, Spain), using a UV–vis spectrophotometer model V-530 (Jasco Co. Ltd., Tokyo, Japan).

#### Wine volatile composition (SPME–GC–MS)

Wine volatile profiles were analyzed after one and four months of storage by SPME–GC–MS, using the same equipment described above. Twenty-five (25) mL of wine were introduced in 50 mL amber glass vials and sealed with PTFE/silicon septa. SPME was operated at 40 °C for 15 min, using a 2 cm length 50/30 µm DVB/Carboxen/PDMS fiber (Supelco, Bellefonte, PA, USA). GC separation was carried out as described by Comuzzo et al*.* (Comuzzo et al. 2012).

### Statistical analysis

All the results were averages of at least three measurements taken from three experiment replications. One-way ANOVA and Tukey HSD test were carried out on the values found for the different parameters analyzed, as well as on the absolute areas of the volatile compounds detected by SPME–GC–MS. Significant differences were considered at p < 0.05. Concerning SPME–GC–MS analyses, the aroma compounds detected in the headspace of the freeze-dried YD powders were grouped, sample by sample, on the basis of their chemical class and the total absolute area obtained for each group was subjected to ANOVA and Principal Component Analysis (PCA). All the elaborations were carried out by the software Statistica for Windows (StatSoft, Tulsa, OK, USA), Version 8.0.

## Results and Discussion

### Release of soluble molecules in wine-like solution

As previously reported (Comuzzo et al. 2015b), HPH favored the release of soluble compounds from yeast (Table 1). The amounts of total colloids released by HPH-YD in wine-like medium were comparable (even if averagely slightly higher) with those found for COMM and T-YD samples. The solubilization of amino acids by the three products was also similar, but the concentrations detected were very low, confirming previous findings (Comuzzo et al. 2015b). Contrary, the three YDs behaved differently concerning the ability to release GSH in model wine. In accordance to what declared by the supplier, COMM showed the highest GSH content, followed by HPH-YD. Contrary, GSH was not detected in T-YD samples, presumably for the high temperatures reached during the production process of these preparations.

**Table 1:** Soluble compounds released by YDs in wine-like solution. Data are means and standard deviations (SD) of three repeated trials; different letters within the same column mark significant differences according to ANOVA and Tukey HSD test at p < 0.05. See the text for abbreviations

	Total colloids (mg g^−1^)	Free amino acids (mg g^−1^)	Glutathione (mg g^−1^)
	Mean ± SD	Mean ± SD	Mean ± SD
HPH-YD	194 ± 7a	6 ± 1a	6 ± 1b
T-YD	185 ± 11a	4 ± 0a	0^a^ ± 0a
COMM	144 ± 38a	3 ± 1a	9 ± 0c

^a^not detected

A suitable concentration of glucidic colloids and a high level of GSH in YD products are suitable features for their use during white wine ageing. Indeed, colloids are able to interact with aroma compounds modulating their volatility and improving wine organoleptic characteristics (Comuzzo et al. 2011; Pozo-Bayón et al. 2009c); GSH plays a positive role for its antioxidant capacity and protective effect on wine aroma (Rodríguez-Bencomo et al. 2014).

Based on these considerations, HPH may represent an interesting perspective for the production of yeast derivatives for wine ageing. The product obtained in the current study was able to release soluble colloids comparably to the commercial product tested (average concentration was even slightly higher); moreover, HPH led to a greater GSH content with respect to thermolysis and for this reason HPH-YD appeared an interesting product to be used as a complement for reducing sulfur dioxide addition during wine storage.

### Volatile composition of YDs

**Fig. 1:**
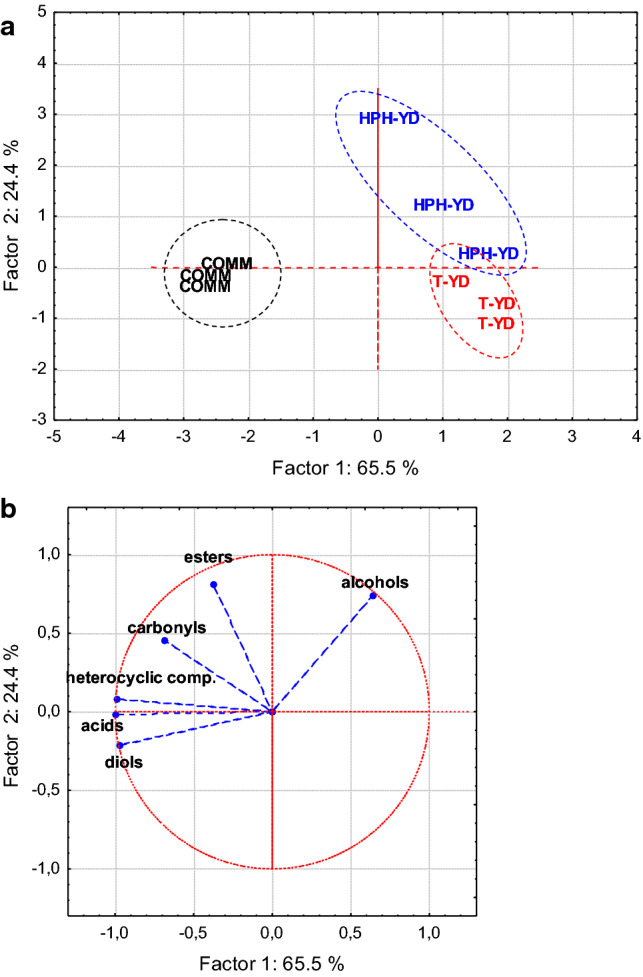
Results of PCA carried out on the absolute areas of the volatile compounds detected in the YD powders and grouped by chemical class. Projections of cases (samples) (**a**) and variables (chemical classes) (**b**) on the factor-plane are reported. See the text for abbreviations

Thirty-five volatile compounds were tentatively identified in the headspace of the freeze-dried powders (Online Resource – Table S1). Compounds were grouped by chemical class and the results of PCA and ANOVA analysis are reported in Fig. 1 and in Online Resource (Table S2) respectively.

Concerning PCA results, the three repetitions analyzed for each YD are clustered quite clearly in Fig. 1a, grouping themselves on the basis of product typology (HPH-YD, T-YD and COMM). The commercial product (COMM) differed from the thermally and HPH-processed YDs because of a higher content of diols, fatty acids and heterocyclic compounds; the reasons of such differences may be connected with the production technology of the three YDs, as well as with the yeast strain used. Diols have a fermentative origin, being normal products of yeast metabolism (Di Stefano et al. 1988). Fatty acids are also produced by yeasts (Luan et al. 2018), but some short-chain fatty acids (e.g. 2-methylpropanoic and 3-methylbutanoic) may also derive from the oxidation of Strecker aldehydes or amino acid degradation during Maillard reaction (Ames and Mc Leod 1985). These compounds were detected as some of the major components of YDs (Comuzzo et al. 2006) and they may be responsible of off-flavors in wine (Swiegers and Pretorius 2005), particularly when YDs are added in high amounts and for long contact times (Comuzzo et al. 2006; Pozo-Bayón et al. 2009b). Finally, heterocyclic compounds include 2-furaldehyde, 2-furanmethanol, alkyl-pyrazines, and γ-butyrolactone. While γ-butyrolactone is normally produced during alcoholic fermentation (Lopez de Lerma et al. 2012), the other compounds are generally associated with the behavior of Maillard reaction (Ames and Mc Leod 1985; Nagodawithana 1992), which takes place during the manufacturing of YDs, in particular during thermal inactivation treatments and during the final dehydration of the products (by spray-drying or drum-drying).

Overall, T-YD and HPH-YD resulted to be less characterized by such volatile compounds with respect to COMM. As already mentioned, this difference might be connected with the yeast strain and the technology used for their production, but it may represent a positive factor, in virtue of a presumed lower capacity to release exogenous aromas and off-flavors in wine. Contrary, HPH-YD showed averagely a higher concentration of alcohols and esters (Online Resource, Table S2). These groups of molecules are typically released in wine during yeast autolysis (Alexandre and Guilloux-Benatier 2006); esters, in particular might give a positive contribution to wine volatile profile, because of their typical pleasant and fruity aroma (Ribéreau-Gayon et al. 2006).

### Effect of YD supplementation on wine aroma evolution

As reported in literature, the effects of YDs on wine aroma are the result of a complex interaction connected with three main aspects: i) the release of aroma compounds from the powders into the wine, ii) the retention of wine volatile compounds onto the insoluble fraction of YDs and iii) the ability of the colloidal fraction released by these products to modulate the volatility of wine aroma molecules (Comuzzo et al. 2012). These effects may depend on several factors, such as the kind of YD, its dosage, the contact time, the tasting temperature, as well as the composition and typology of the wine (Comuzzo et al. 2006, 2011; Pozo-Bayón et al. 2009a, b).

In the present experiment, the three YDs were added to a white wine to assess their potential impact on the evolution of its aroma profile. Twenty-four volatile compounds were tentatively identified by SPME–GC–MS in the headspace of the samples analyzed, one and four months after YD supplementation (Online Resource – Table S3). Table 2 reports the semi-quantitative results obtained one month after the treatment. Significant differences among samples were detected for a limited number of compounds, mainly diols and esters. Diols (2,3-butanediol and 1,2-propanediol) were more abundant in the wines treated with HPH-YD, but they generally give a minor contribution to wine aroma (Ribéreau-Gayon et al. 2006). Conversely, the behavior of esters appeared more interesting. The addition of the three YD products determined a diminution of the headspace concentration of some of the most important wine esters: ethyl acetate (EAc), 3-methyl-1-butanol acetate (3-MeBA), ethyl hexanoate (EHex) and ethyl decanoate (EDec) were averagely less abundant in the samples treated with YDs than in control wine. This seems mostly connected with the ability of YDs to bind wine volatile compounds (Pozo-Bayón et al. 2009c; Comuzzo et al. 2011), while their capacity to increase the volatility of certain aroma molecules (Comuzzo et al. 2011) was not observed in the present experiment. According to ANOVA, such diminution was statistically significant compared to the control, only for T-IDY and COMM (including EAc, 3-MeBA and EHex for the former and EDec for the latter). This highlights, for these two products, a higher binding capacity towards wine esters with respect to HPH-YD and, potentially, a lower global impact of the latter on wine aroma.

**Table 2:** Volatile compounds detected in the headspace of the Chardonnay wines treated with the YDs (400 mg L^−1^ each) and in their controls (untreated wines), after one month of storage. Data are means and standard deviations (SD) of three repeated trials; different letters within the same row mark significant differences according to ANOVA and Tukey HSD test at p < 0.05. See the text for abbreviations

Compound	*Rt*^a^	Absolute area/10^6^			
		Control	HPH-YD	T-YD	COMM
		Mean ± SD	Mean ± SD	Mean ± SD	Mean ± SD
*Alcohols*					
Ethanol	3.4	888 ± 31a	975 ± 80a	951 ± 41a	939 ± 30a
2-methyl-1-propanol	6.5	31 ± 10a	24 ± 1a	29 ± 13a	30 ± 9a
2- and 3-methyl-1-butanol	9.5	1043 ± 64a	1041 ± 31a	1035 ± 49a	1056 ± 85a
1-hexanol	13.9	134 ± 1a	142 ± 1b	138 ± 6ab	140 ± 3ab
2-phenylethanol	29.8	97 ± 3a	107 ± 7a	108 ± 1a	101 ± 13a
*Acids*					
2-methylpropanoic acid	20.4	2 ± 1a	1 ± 1a	2 ± 0a	2 ± 0a
Butanoic acid	22.1	4 ± 2a	2 ± 1a	4 ± 1a	2 ± 0a
3-methylbutanoic acid	23.4	3 ± 1a	2 ± 1a	3 ± 0a	3 ± 0a
Hexanoic acid	28.2	134 ± 7a	133 ± 8a	130 ± 4a	128 ± 16a
Octanoic acid	33.7	345 ± 20a	352 ± 23a	376 ± 30a	348 ± 69a
Decanoic acid	38.6	89 ± 12a	67 ± 10a	73 ± 11a	57 ± 18a
*Diols*					
2,3-butanediol	19.7	57 ± 8a	106 ± 29b	61 ± 17ab	54 ± 8a
1,2-propanediol	20.8	16 ± 2ab	36 ± 14b	15 ± 7a	14 ± 1a
*Esters*					
Ethyl acetate	2.8	422 ± 38b	348 ± 57ab	320 ± 21a	339 ± 17ab
Ethyl butanoate	4.9	61 ± 4a	40 ± 31a	23 ± 10a	27 ± 6a
3-methyl-1-butanol acetate	6.8	1112 ± 72b	605 ± 233ab	467 ± 157a	693 ± 257ab
Ethyl hexanoate	10.1	1408 ± 59b	972 ± 312ab	850 ± 172a	976 ± 140ab
Ethyl lactate	13.4	18 ± 8a	22 ± 2a	22 ± 1a	22 ± 3a
Ethyl decanoate	22.7	1517 ± 390b	1055 ± 171ab	1131 ± 188ab	819 ± 132a
Diethyl succinate	23.5	15 ± 1a	16 ± 1a	16 ± 1a	17 ± 2a
2-phenylethyl acetate	27.2	40 ± 1a	40 ± 3a	43 ± 4a	41 ± 9a
*Others*					
3-hydroxy-2-butanone (acetoin)	11.5	13 ± 4a	21 ± 1a	20 ± 5a	16 ± 3a
Benzaldehyde	18.7	5 ± 1a	4 ± 1a	4 ± 0a	4 ± 1a

^a^*Rt*, retention time

The volatile composition of the wines after four months of storage is reported in Table 3. Significant variations according to ANOVA were found for 3-methylbutanoic acid (3-MeB) and for some esters. The former (3-MeB) significantly increased in all the wines treated with YDs. This compound is one of the most representative components of the volatile composition of YDs (Comuzzo et al. 2012) and presumably the increase of its concentration in YD-treated wines might be connected with its release from the powders. It is interesting to observe that 3-MeB did not increase with YD supplementation after one month of storage (Table 2), meaning that longer contact times are needed for determining the solubilization of certain compounds from the powders, in agreement with the findings of Pozo-Bayón et al*.* (Pozo-Bayón et al. 2009b).

**Table 3:** Volatile compounds detected in the headspace of the Chardonnay wines treated with the YDs (400 mg L^−1^ each) and in their controls (untreated wines), after four months of storage. Data are means and standard deviations (SD) of three repeated trials; different letters within the same row mark significant differences according to ANOVA and Tukey HSD test at p < 0.05. See the text for abbreviations. ±

Compound	*Rt*^a^	Absolute area/10^6^			
		Control	HPH-YD	T-YD	COMM
		Mean ± SD	Mean ± SD	Mean ± SD	Mean ± SD
*Alcohols*					
Ethanol	3.4	1284 ± 43a	1326 ± 79a	1310 ± 44a	1292 ± 23a
2-methyl-1-propanol	6.5	15 ± 3a	13 ± 2a	17 ± 9a	13 ± 1a
2- and 3-methyl-1-butanol	9.5	953 ± 20a	1026 ± 72a	988 ± 31a	970 ± 49a
1-hexanol	13.9	131 ± 2a	125 ± 8a	130 ± 2a	129 ± 10a
2-phenylethanol	29.8	106 ± 7a	102 ± 9a	100 ± 11a	106 ± 6a
*Acids*					
2-methylpropanoic acid	20.4	3 ± 0a	4 ± 1a	3 ± 0a	3 ± 0a
Butanoic acid	22.1	3 ± 1a	3 ± 1a	3 ± 0a	3 ± 1a
3-methylbutanoic acid	23.4	0 ± 0a	4 ± 1b	5 ± 1b	5 ± 0b
Hexanoic acid	28.2	107 ± 8a	113 ± 10a	104 ± 12a	110 ± 9a
Octanoic acid	33.7	306 ± 35a	318 ± 41a	279 ± 35a	306 ± 39a
Decanoic acid	38.6	76 ± 7a	64 ± 12a	54 ± 9a	53 ± 10a
*Diols*					
2,3-butanediol	19.7	102 ± 16a	91 ± 17a	94 ± 18a	78 ± 17a
1,2-propanediol	20.8	34 ± 10a	31 ± 9a	29 ± 9a	22 ± 4a
*Esters*					
Ethyl acetate	2.8	436 ± 15a	361 ± 100a	381 ± 12a	405 ± 37a
Ethyl butanoate	4.9	48 ± 3a	40 ± 14a	35 ± 5a	39 ± 10a
3-methyl-1-butanol acetate	6.8	474 ± 73a	296 ± 140a	275 ± 74a	395 ± 158a
Ethyl hexanoate	10.1	1534 ± 37a	1123 ± 397a	1240 ± 62a	1236 ± 34a
Hexyl acetate	11.2	26 ± 13a	9 ± 3a	5 ± 2a	17 ± 9a
Ethyl lactate	13.4	55 ± 2a	66 ± 4b	68 ± 4b	68 ± 5b
Ethyl decanoate	22.7	1682 ± 106b	986 ± 90a	1147 ± 119a	931 ± 98a
Diethyl succinate	23.5	28 ± 3b	23 ± 2ab	21 ± 2a	23 ± 3ab
2-phenylethyl acetate	27.2	19 ± 4a	22 ± 5a	16 ± 2a	23 ± 2a
*Others*					
3-hydroxy-2-butanone (acetoin)	11.5	22 ± 7a	22 ± 3a	24 ± 3a	22 ± 3a
Benzaldehyde	18.7	3 ± 1a	3 ± 0a	3 ± 0a	3 ± 0a

^a^*Rt*: retention time

Among esters, significant variations after four months were found for EDec, and the two ageing esters ethyl lactate (ELac) and diethyl succinate (DESu) (Table 3). The former is a fermentative ester, produced by yeasts during alcoholic fermentation (Ribéreau-Gayon et al. 2006). As it was after one month (Table 2), YD treatments produced a significant decrease of EDec concentration, as well as of the average absolute areas of the other esters analyzed. Concerning ageing esters, DESu also showed an average decrease with YD addition. Contrary, ELac slightly (but significantly) increased in presence of YDs. The genesis of this compound is reported to be linked to malolactic fermentation and its concentration normally rises during wine ageing (Ribéreau-Gayon et al. 2006). Its augmentation in YD-treated wines might be connected with the claimed “salting out effects” described for these products (Pozo-Bayón et al. 2009a), i.e. the ability of the colloidal fraction released by YDs, to enhance the volatility of certain aroma compounds.

Basing on these results, HPH-YD determined minor modifications on wine volatile composition for short contact times and behaved similarly to the commercial preparation tested, giving an even lower impact on wine aroma and a lower retention of volatile compounds with respect to the thermolysate. When contact time increased the differences among products tended to disappear and the concentration of compounds typically found in YDs increased in the treated wines.

### Effect of YDs on wine color

**Fig. 2:**
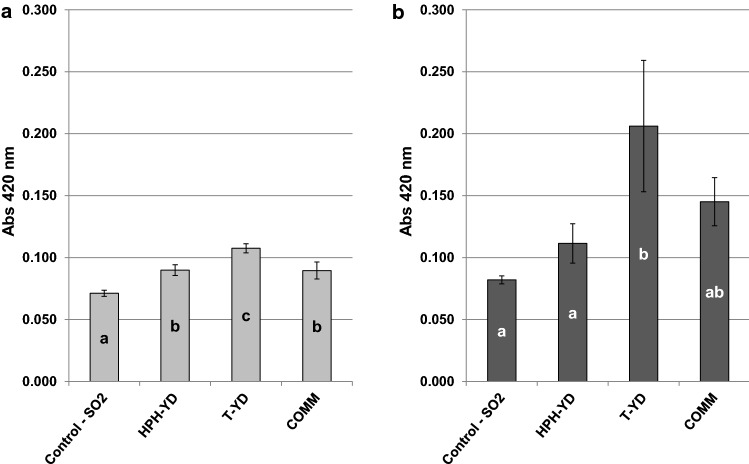
Absorbance at 420 nm measured for the Chardonnay wines treated with the YDs (400 mg L^−1^ each) and for their controls (wine + SO_2_ 50 mg L^−1^), after one (**a**) and four months (**b**) of storage. Data are means of three repeated trials and different letters within the same graph mark significant differences according to ANOVA and Tukey HSD test at p < 0.05. Vertical bars represent standard deviation. See the text for abbreviations

Despite few papers discussed the antioxidant capacity of YDs from the scientific point of view (Rodríguez-Bencomo et al. 2014; Comuzzo et al. 2015a), several commercial preparations are available to winemakers for this specific scope. In the present experiment, the effects of the supplementation with the three YDs on wine color changes during ageing were tested in comparison with sulfur dioxide addition (50 mg L^−1^), with the aim of assessing the capacity of YDs to maintain the initial bright straw yellow color of the wines. Results are reported in Fig. 2.

All YDs determined a more intense browning with respect to sulfiting, in particular after four months of conservation (Fig. 2b). T-YD was the less performing additive, allowing an intense color development even after such a relatively low period of storage (four months). Contrary, HPH-YD was the most efficient preparation, limiting wine color increments during four months and behaving more similarly to SO_2_ (Fig. 2b). COMM also showed good performances, with intermediate results between the other two products.

The less intense browning achieved with COMM and HPH-YD, with respect to T-YD, might be related to their higher GSH content (Table 1). However, it is interesting to note that the effect of these two YDs on wine color development was not proportional to their initial GSH concentration. Indeed, the former showed the highest GSH amount in the powder (Table 1), but the latter allowed a slightly better color evolution (Fig. 2b), better preserving the value of Abs 420 nm initially measured in the wine. This may confirm that GSH is not the sole responsible of the antioxidant capacity of YDs (Rodríguez-Bencomo et al. 2014), though the tripeptide might be anyhow a possible marker of oxidative stress during YDs manufacturing.

Finally, the data obtained also confirmed that sulfur dioxide remains the most efficient additive in preventing wine browning; in the sulfited samples, wine color only slightly changed, moving from one to four months of ageing (Fig. 2).

**Fig. 3:**
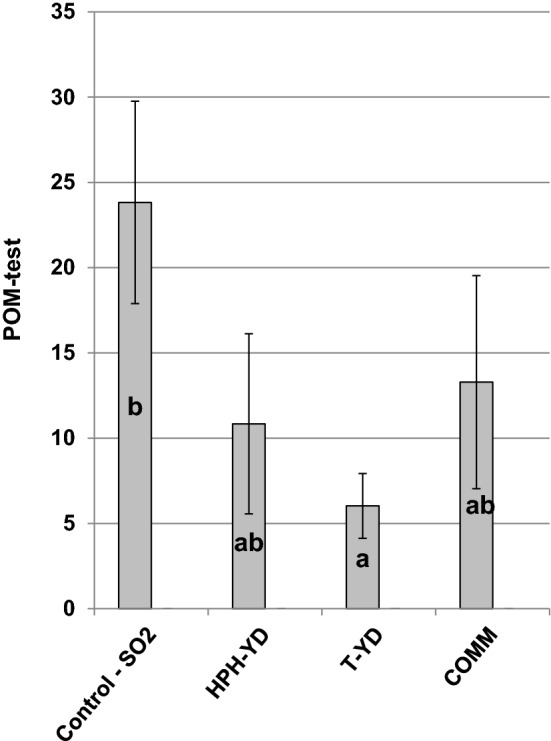
POM-test values detected for the Chardonnay wines treated with the YDs (400 mg L^−1^ each) and for their controls (wine + SO_2_ 50 mg L^−1^) after one month of storage. Data are means of three repeated trials and different letters mark significant differences according to ANOVA and Tukey HSD test at p < 0.05. Vertical bars represent standard deviation. See the text for abbreviations

The results obtained for the POM-test (browning assay) confirmed the behaviors observed for wine color. One month after the treatment (Fig. 3), sulfiting was the most performing practice for preserving wine phenolics and oxidation potential (highest POM-test value). COMM and HPH-YD were less efficient, while T-YD gave the lowest POM-test index, confirming its minor capacity to protect wine phenolic compounds against oxidation. POM-test was not detectable after four months of storage, denoting that the wine had probably reached its overall ageing potential.

## Conclusion

In conclusion, HPH demonstrated to be a suitable and interesting technique for the production of yeast derivatives for winemaking. The autolysate produced in the present study behave similarly to the commercial product tested, concerning the release of glucidic colloids and the antioxidant capacity, with a limited impact on the aromatic composition of the treated wines, and an overall better enological performance with respect to the preparation obtained by thermally-assisted autolysis.

It should also be noted that the commercial product used in the present experiment has been obtained by thermal inactivation and this certainly had an impact on its aroma composition and GSH content, as demonstrated in the current experiment and in previous publications. To solve this problem, Rodríguez-Bencomo and colleagues report that YDs available commercially may be enriched in GSH even if “it is still not clear whether exogenous GSH enrichment is allowed during the manufacturing process” (Rodríguez-Bencomo et al. 2014). For this reason, HPH could represent a suitable technique to preserve the natural antioxidant potential of commercial YDs and their ability to protect wine aroma, without exogenous GSH supplementation.

Of course, further optimizations are necessary to improve the process before its scale-up, in particular focusing on the reduction of the number of passes. This will make this specific application of HPH sustainable from the economical point of view, in compliance with the OIV recommendations for the characteristics of YD products for winemaking.

## Electronic supplementary material

Below is the link to the electronic supplementary material.

Supplementary file1 (DOC 183 kb)
