# Supplementary material for: Effect of a yeast autolysate produced by high pressure homogenization on white wine evolution during ageing
Source: J Food Sci Technol. 2020 Oct 24;58(10):4045–54. doi: 10.1007/s13197-020-04867-8 (PMC8357859; doi:10.1007/s13197-020-04867-8)
Supplement: Supplementary file 1 — Supplementary file1 (DOC 183 kb) [file 13197_2020_4867_MOESM1_ESM.doc]

Journal of Food Science and Technology

Original Research Paper

# Effect of a yeast autolysate produced by high pressure homogenization on white wine evolution during ageing

**Online Resource**

**Supplementary Material**

**Table S1 Volatile compounds tentatively identified by SPME-GC-MS in the headspace of the YD powders.**

| **Compound** | | **Ri^a^** | **Ri_(lit)_^b^** | **Reference** | **IM^c^** |
| --- | --- | --- | --- | --- | --- |
| *Alcohols* | |  |  |  |  |
| etanol | | 936 | 929 | (Acree and Arn 2019) | MS, RI |
| 2-methyl-1-propanol | | 1093 | 1096 | (Lopez et al. 1999) | MS, RI |
| 1-butanol | | 1149 | 1145 | (Baek and Cadwallader 1999) | MS, RI, S |
| 2- and 3-methyl-1-butanol | | 1212 | 1210 | (Baek and Cadwallader 1999) | MS, RI, S |
| 1-hexanol | | 1359 | 1359 | (Lopez et al. 1999) | MS, RI, S |
| 2-butoxyethanol | | 1400 |  |  | MS |
| 2-ethyl-1-hexanol | | 1493 | 1490 | (Madruga and Mottram 1998) | MS, RI, S |
| 2-phenylethanol | | 1902 | 1922 | (Baek and Cadwallader 1999) | MS, RI, S |
|  | |  |  |  |  |
| *Acids* | |  |  |  |  |
| acetic acid | | 1448 | 1451 | (Baek and Cadwallader 1999) | MS, RI, S |
| propanoic acid | | 1530 | 1528 | (Münch et al. 1997) | MS, RI, S |
| 2-methylpropanoic acid | | 1567 | 1548 | (Münch et al. 1997) | MS, RI, S |
| butanoic acid | | 1627 | 1612 | (Münch et al. 1997) | MS, RI, S |
| 3-methylbutanoic acid | | 1669 | 1672 | (Baek and Cadwallader 1999) | MS, RI, S |
| hexanoic acid | | 1848 | 1854 | (Lopez et al. 1999) | MS, RI, S |
| 2-ethylhexanoic acid | | 1947 | 1974 | (Welke et al. 2012) | MS, RI |
|  | |  |  |  |  |
| *Carbonyls* | |  |  |  |  |
| hexanal | | 1080 | 1084 | (Jennings and Shibamoto 1980) | MS, RI, S |
| heptanal | | 1182 | 1184 | (Goodner 2008) | MS, RI |
| 3-hydroxy-2-butanone (acetoin) | | 1281 | 1290 | (Baek and Cadwallader 1999) | MS, RI, S |
| 1-hydroxy-2-propanone (acetylcarbinol) | | 1290 | 1300 | (Gonzalez-Rios et al. 2007) | MS, RI |
| 6-methyl-5-hepten-2-one | | 1333 | 1336 | (Comuzzo et al. 2006) | MS, RI |
| nonanal | | 1388 | 1396 | (Vichi et al. 2003) | MS, RI |
|  | |  |  |  |  |
| *Diols* | |  |  |  |  |
| 2,3-butandiol | | 1545 | 1545 | (Baek and Cadwallader 1999) | MS, RI |
| 1,2-propandiol | | 1582 | 1594 | (Wong and Bernhard 1988) | MS, RI |
| 1,4-butandiol | | 1924 | 1861 | (Jennings and Shibamoto 1980) | MS |
|  | |  |  |  |  |
| *Esters* | |  |  |  |  |
| ethyl acetate | |  |  |  | MS |
| ethyl octanoate | | 1432 | 1435 | (Baek and Cadwallader 1999) | MS, RI, S |
| ethyl 3-hydroxybutanoate | | 1514 |  |  | MS |
| ethyl decanoate | | 1635 | 1634 | (Lopez et al. 1999) | MS, RI, S |
|  | |  |  |  |  |
| *Heterocyclic compounds* | |  |  |  |  |
| 2-furaldehyde (furfural) | | 1452 | 1475 | (Lopez et al. 1999) | MS, RI, S |
| dihydro-2(3*H*)-furanone (γ-butyrolactone) | | 1618 | 1632 | (Jennings and Shibamoto 1980) | MS, RI, S |
| 2-furanmethanol | | 1655 | 1673 | (Comuzzo et al. 2006) | MS, RI |
| 5,6-dihydro-2*H*-pyran-2-one | | 1688 |  |  | MS |
| 2-ethyl-6-methylpyrazine | | 1382 | 1384 | (Comuzzo et al. 2006) | MS, RI |
| 2,3,5-trimethylpyrazine | | 1401 | 1408 | (Comuzzo et al. 2006) | MS, RI |
| 3-ethyl-2,5-dimethylpyrazine | | 1445 | 1464 | (Comuzzo et al. 2006) | MS, RI |
| a | Ri: Calculated linear retention index | | | | |
| b | Ri_(lit)_: Linear retention index from literature | | | | |
| c | IM: identification method:  **S:** comparison of mass spectra and retention time with those of standard compounds; **RI:** comparison of order of elution with those reported in literature; **MS:** comparison of mass spectra with those reported in Wiley 6 and NIST 107 mass spectrum libraries | | | | |

**Table S2** Results of ANOVA and Tukey HSD test carried out on the absolute areas of the volatile compounds detected in the YD powders and grouped by their chemical class. Data are means and standard deviations (SD) of three repeated trials; different letters within the same row mark significant differences at p < 0.05. See the text for abbreviations.

| Chemical class | Absolute area / 10^6^ | | | | | | | | | | | |
| --- | --- | --- | --- | --- | --- | --- | --- | --- | --- | --- | --- | --- |
|  | HPH-YD | | | | T-YD | | | | COMM | | | |
|  | Mean | + | SD |  | Mean | + | SD |  | Mean | + | SD |  |
| Alcohols | 1907 | + | 424 | **c** | 860 | + | 88 | **b** | 86 | + | 14 | **a** |
| Acids | 420 | + | 291 | **a** | 90 | + | 18 | **a** | 1959 | + | 54 | **b** |
| Carbonyls | 37 | + | 11 | **a** | 27 | + | 14 | **a** | 43 | + | 6 | **a** |
| Diols | 21 | + | 11 | **a** | 6 | + | 1 | **a** | 724 | + | 17 | **b** |
| Esters | 43 | + | 29 | **a** | 6 | + | 8 | **a** | 35 | + | 15 | **a** |
| Heterocyclic compounds | 14 | + | 7 | **a** | 7 | + | 1 | **a** | 36 | + | 2 | **b** |

**Table S3 Volatile compounds tentatively identified by SPME-GC-MS in the headspace of wines.**

| **Compound** | | **Ri^a^** | **Ri_(lit)_^b^** | **Reference** | **IM^c^** |
| --- | --- | --- | --- | --- | --- |
| *Alcohols* | |  |  |  |  |
| ethanol | | 946 | 929 | (Acree and Arn 2019) | MS, RI |
| 2-methyl-1-propanol | | 1.105 | 1.096 | (Lopez et al. 1999) | MS, RI |
| 2- and 3-methyl-1-butanol | | 1.214 | 1210 | (Baek and Cadwallader 1999) | MS, RI, S |
| 1-hexanol | | 1.356 | 1.359 | (Lopez et al. 1999) | MS, RI, S |
| 2-phenylethanol | | 1.900 | 1.922 | (Baek and Cadwallader 1999) | MS ,RI, S |
|  | |  |  |  |  |
| *Acids* | |  |  |  |  |
| 2-methylpropanoic acid | | 1.567 | 1.548 | (Münch et al. 1997) | MS, RI, S |
| butanoic acid | | 1.627 | 1.612 | (Münch et al. 1997) | MS, RI, S |
| 3-methylbutanoic acid | | 1.669 | 1.672 | (Baek and Cadwallader 1999) | MS, RI, S |
| hexanoic acid | | 1.839 | 1.852 | (Lopez et al. 1999) | MS, RI, S |
| octanoic acid | | 2.053 | 2.060 | (Lopez et al. 1999) | MS, RI, S |
| decanoic acid | | 2.268 | 2.229 | (Lopez et al. 1999) | MS |
|  | |  |  |  |  |
| *Diols* | |  |  |  |  |
| 2,3-butanediol | | 1.540 | 1.545 | (Baek and Cadwallader 1999) | MS, RI |
| 1,2-propanediol | | 1.577 | 1594 | (Wong and Bernhard 1988) | MS, RI |
|  | |  |  |  |  |
| *Esters* | |  |  |  |  |
| ethyl acetate | |  |  |  | MS |
| ethyl butanoate | | 1.036 | 1.034 | (Baek and Cadwallader 1999) | MS, RI |
| 3-methyl-1-butanol acetate | | 1.117 | 1.128 | (Baek and Cadwallader 1999) | MS, RI |
| ethyl hexanoate | | 1.233 | 1.234 | (Baek and Cadwallader 1999) | MS, RI, S |
| hexyl acetate | | 1.269 | 1.275 | (Baek and Cadwallader 1999) | MS, RI |
| ethyl lactate | | 1.340 | 1.358 | (Acree and Arn 2019) | MS, RI |
| ethyl decanoate | | 1.642 | 1.634 | (Lopez et al. 1999) | MS, RI, S |
| diethyl succinate | | 1.670 | 1.642 | (Jennings and Shibamoto 1980) | MS, RI |
| 2-phenylethyl acetate | | 1.801 | 1.820 | (Baek and Cadwallader 1999) | MS, RI |
|  | |  |  |  |  |
| *Others* | |  |  |  |  |
| 3-hydroxy-2-butanone (acetoin) | | 1.279 | 1.290 | (Baek and Cadwallader 1999) | MS, RI, S |
| benzaldehyde | | 1.506 | 1.528 | (Baek and Cadwallader 1999) | MS, RI, S |
| a | Ri: Calculated linear retention index | | | | |
| b | Ri_(lit)_: Linear retention index from literature | | | | |
| c | IM: identification method: **S:** comparison of mass spectra and retention time with those of standard compounds; **RI:** comparison of order of elution with those reported in literature; **MS:** comparison of mass spectra with those reported in Wiley 6 and NIST 107 mass spectrum libraries | | | | |

**Additional literature for GC-MS identification**

Acree T, Arn H (2019) Flavornet and human odor space. Gas chromatography - olfactometry (GCO) of natural products. http//www.flavornet.org/flavornet.html. Accessed 15 April 2019

Baek H, Cadwallader K (1999) Contribution of free and glycosidically bound volatile compounds to the aroma of muscadine grape juice. J Food Sci 64:441–444

Comuzzo P, Tat L, Tonizzo A, Battistutta F (2006) Yeast derivatives (extracts and autolysates) in winemaking: Release of volatile compounds and effects on wine aroma volatility. Food Chem 99:217–230

Gonzalez-Rios O, Suarez-Quiroz M, Boulanger R, et al (2007) Impact of “ecological” post-harvest processing on coffee aroma: II. Roasted coffee. J Food Compos Anal 20:297–307

Goodner K (2008) Practical retention index models of OV-101, DB-1, DB-5, and DB-Wax for flavor and fragrance compounds. LWT - Food Sci Technol 41:951–958

Jennings W, Shibamoto T (1980) Qualitative analysis of flavor and fragrance volatiles by glass capillary gas chromatography. Academic Press, New York

Lopez R, Ferreira V, Hernandez P, Cacho J (1999) Identification of impact odorants of young red wines made with Merlot, Cabernet Sauvignon and Grenache grape varieties: a comparative study. J Sci Food Agric 79:1461–1467

Madruga M, Mottram D (1998) The effect of pH on the formation of volatilecompounds by heating a model system containing 5’-IMP and cysteine. J Brazilian Chem Soc 9:261–271

Münch P, Hofmann T, Schieberle P (1997) Comparison of key odorants generated by thermal treatment of commercial and self-prepared yeast extracts: influence of the amino acid composition on odorant formation. J Agric Food Chem 45:1338–1344

Vichi S, Pizzale L, Conte L, et al (2003) Solid-phase microextraction in the analysis of virgin olive oil volatile fraction: modifications induced by oxidation and suitable markers of oxidative status. J Agric Food Chem 51:6564–6571

Welke J, Manfroi V, Zanus M, et al (2012) Characterization of the volatile profile of Brazilian Merlot wines through comprehensive two dimensional gas chromatography time-of-flight mass spectrometric detection. J Chromatogr A 1226:124–139

Wong J, Bernhard R (1988) Effect of nitrogen source on pyrazine formation. J Agric Food Chem 36:123–129
